# Using the Medical Research Council framework for development and evaluation of complex interventions in a low resource setting to develop a theory-based treatment support intervention delivered via SMS text message to improve blood pressure control

**DOI:** 10.1186/s12913-017-2808-9

**Published:** 2018-01-23

**Authors:** Kirsten Bobrow, Andrew Farmer, Nomazizi Cishe, Ntobeko Nwagi, Mosedi Namane, Thomas P. Brennan, David Springer, Lionel Tarassenko, Naomi Levitt

**Affiliations:** 1 Chronic Disease Initiative for Africa, Cape Town, South Africa; 2 Division of Diabetic Medicine and Endocrinology, Department of Medicine, University of Cape Town, Cape Town, South Africa; 3 Primary Care Clinical Trials Unit, Nuffield Department of Primary Care Health Sciences, University of Oxford, Oxford, UK; 4 Women’s Health Research Unit, School of Public Health & Family Medicine, University of Cape Town, Cape Town, South Africa; 5 Western Cape Province Department of Health, Cape Town, South Africa; 6 Institute of Biomedical Engineering, Department of Engineering Science, University of Oxford, Oxford, UK; 7 Nuffield Department of Primary Care Health Sciences, Radcliffe Observatory Quarter, University of Oxford, Oxford, OX2 6GG UK

**Keywords:** Intervention development, MRC framework, Adherence, Health care, Self-management, Behaviour modification

## Abstract

**Background:**

Several frameworks now exist to guide intervention development but there remains only limited evidence of their application to health interventions based around use of mobile phones or devices, particularly in a low-resource setting. We aimed to describe our experience of using the Medical Research Council (MRC) Framework on complex interventions to develop and evaluate an adherence support intervention for high blood pressure delivered by SMS text message. We further aimed to describe the developed intervention in line with reporting guidelines for a structured and systematic description.

**Methods:**

We used a non-sequential and flexible approach guided by the 2008 MRC Framework for the development and evaluation of complex interventions.

**Results:**

We reviewed published literature and established a multi-disciplinary expert group to guide the development process. We selected health psychology theory and behaviour change techniques that have been shown to be important in adherence and persistence with chronic medications. Semi-structured interviews and focus groups with various stakeholders identified ways in which treatment adherence could be supported and also identified key features of well-regarded messages: polite tone, credible information, contextualised, and endorsed by identifiable member of primary care facility staff. Direct and indirect user testing enabled us to refine the intervention including refining use of language and testing of interactive components.

**Conclusions:**

Our experience shows that using a formal intervention development process is feasible in a low-resource multi-lingual setting. The process enabled us to pre-test assumptions about the intervention and the evaluation process, allowing the improvement of both. Describing how a multi-component intervention was developed including standardised descriptions of content aimed to support behaviour change will enable comparison with other similar interventions and support development of new interventions. Even in low-resource settings, funders and policy-makers should provide researchers with time and resources for intervention development work and encourage evaluation of the entire design and testing process.

**Trial registration:**

The trial of the intervention is registered with South African National Clinical Trials Register number (SANCTR DOH-27-1212-386; 28/12/2012); Pan Africa Trial Register (PACTR201411000724141; 14/12/2013); ClinicalTrials.gov (NCT02019823; 24/12/2013).

## Background

Raised blood pressure is an important and common modifiable risk factor for cardiovascular and related diseases including stroke and chronic kidney disease [1]. Although evidence exists that lowering blood pressure substantially reduces this risk, strategies to achieve sustained blood pressure control are complex. These include modifying a range of behaviours related to health including attending clinic appointments, taking medication regularly and persisting with treatment [2–5].

Mobile communications technology has the potential to support behaviour change and treatment adherence in real time by facilitating remote, interactive, timely access to relevant information, providing context-specific support and prompts to action [6].

Systematic reviews of health behaviour change interventions delivered by mobile phones or devices (m-health) have shown small beneficial effects for some conditions in some settings but results are not consistent [7, 8]. Some though not all trials have shown modest effects on treatment adherence and disease outcomes for m-health interventions among adults living with HIV [9, 10]. Similar results have been found in trials of m-health interventions to support behaviour change for people with high blood pressure, diabetes, and heart disease [11, 12].

Behavioural interventions, including those delivered using m-health technologies are often not systematically developed, specified, or reported [13]. The potential to accumulate evidence of effectiveness and to identify the “active components” in successful m-health interventions depends in part on replication of successful interventions across settings and in part on refining interventions (adding or subtracting elements) using evidence of behaviour change [14]. Adequate descriptions of the theory of the intervention and specific intervention components are needed to extend the evidence base in the field and to facilitate evidence synthesis [15].

Several frameworks are now available to guide intervention development but there is limited evidence of their application to describe the development of m-health interventions particularly in resource constrained settings [16, 17]. The Medical Research Council (MRC) Framework for the development of complex interventions (initially published in 2000 and up-dated in 2008) has been used successfully across disciplines which suggest its flexible, non-linear approach may be usefully applied to the iterative design processes used in the development of new technology-based systems [15, 18–20].

The aim of this paper is to describe our experience of using the 2008 MRC framework to develop and test a theory-based behaviour change intervention to support adherence to high blood pressure treatment delivered by mobile phone text message; to reflect on the benefits and challenges of applying this framework in a resource constrained setting, and to describe the final intervention in line with reporting guidelines for a structured and systematic description [13].

## Methods

**Fig. 1:**
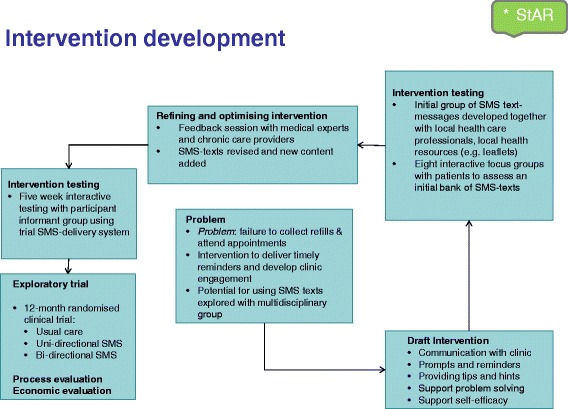
Process of intervention development adapted from Smith et al. [20]

We used a non-sequential, flexible approach guided by the 2008 MRC Framework for the development and evaluation of complex interventions (see Fig. 1) [18]. Table 1 shows the stages of the 2008 MRC framework alongside with the activities we undertook in the development process. Implicit in this development process is the identification of contextual factors that can affect outcomes [21].

**Table 1:** Mapping activities to MRC framework

1	Developing complex intervention		
1.1	Identifying evidence base by reviewing published literature and existing systematic reviews	i	Epidemiology of high blood pressure and its control
		ii	Evidence base on treatment adherence
		iii	Identified existing systematic reviews and trials of interventions delivered by mobile phone
1.2	Identifying and developing appropriate theory	i	Literature review and meeting with stakeholders and experts to decide on theory, behaviour change techniques, and intervention strategies
		ii	Qualitative studies with stakeholder groups to refine content and test delivery system
1.3	Modelling process and outcomes	i	Used causal modelling approach to link determinants of behaviour to behaviours and subsequent health outcomes
2	Assessing feasibility and piloting methods		
2.1	Testing procedures for acceptability, compliance, and intervention delivery	i.	Tested components for feasibility and acceptability
		ii.	Service tested full intervention over 8-week period
2.2	Estimating recruitment and retention	i.	Recruitment from general outpatient department of a large, single primary care facility
		ii.	Review of literature to determine best practice for ongoing retention of trial participants
2.3	Determining sample size	i.	Data from observational studies used to calculate sample size
3	Evaluating complex intervention		
3.1	Assessing effectiveness	i.	Set up a large pilot RCT (South African National Clinical Trials Register DOH-27-1212-386; 28/12/2012; Pan Africa Trial Register PACTR201411000724141; 14/12/2013); ClinicalTrials.gov NCT02019823; 24/12/2013). Primary outcome change in mean systolic blood pressure at 1 year, data on secondary outcomes along hypothesised casual pathway also collected. Usual care group get infrequent non-health related SMS text messages, intervention groups get regular SMS text messages designed to support treatment adherence
3.2	Understanding change processes	i.	Intervention fidelity assessed using message delivery logs and logs of participant contact
		ii.	In final phase of the trial qualitative study of participants, health care workers, and service providers to explore how the intervention might work (necessary pre-requisites), could be optimised, contextual factors, specific key ingredients which could be included in future interventions
3.3	Cost-effectiveness	i.	Data on costs of developing, testing, and delivering intervention as well as health service costs collected and analyses in process
4	Implementation and beyond		
4.1	Dissemination	i.	Peer review publications, conference presentations, public engagement activities, making available tools used to develop and deliver intervention
4.2	Surveillance, monitoring, and long-term outcomes	i.	Consent to access routinely collected health data. If intervention shown to be effective then process and outcome data could inform additional pragmatic trials.

## Results

### Developing a complex intervention

#### Identifying the evidence base

We searched PubMed, Cochrane reviews, and Google for systematic reviews and published original studies from 2000 onwards that were written in English. We used search terms including “mobile health, text-message, adherence, high blood pressure, hypertension and adherence”. We revisited the literature and narrowed the focus of our reviews as development of the intervention progressed. We set up automatic alerts to monitor the relevant literature for updates.

There is some evidence that clinical outcomes for treatment of chronic conditions can be modestly improved through targeting adherence behaviour [4, 22], with a number of trials in hypertension [5, 23–25]. The most effective strategies for improving adherence were complex including combinations of more instructions and health education for patients, disease and treatment-specific adherence counselling, automated and in-person telephone follow-up, and reminders (for pills, appointments, and prescription refills) [22]. In addition some strategies can be costly, for example with case management and pharmacy-based education. These approaches may not be practical in a low-resource setting.

Some studies report that mobile phone messaging interventions may provide benefit in supporting the self-management of long-term illnesses [7, 26], and have the potential to support lifestyle change, including smoking cessation [7, 27]. However randomised trials of the effectiveness of mobile phone messaging in the management of hypertension are few, include additional components (telemonitoring), often focus on high-risk groups such as stroke survivors and renal transplant recipients, and are based in high-resource settings [23, 28–31].

#### Identifying appropriate theory

We set up an expert multi-disciplinary group comprising two specialist general practitioners, two specialist physicians, three biomedical engineers, a health systems researcher, and an epidemiologist. As a team we met formally to agree upon the research problem and the underlying principles guiding the intervention development process. Thereafter we worked in smaller groups to develop the intervention. We maintained written logs of the iterative steps of the intervention development and remained in regular contact with the full group via email and teleconference. When the group met formally we reported on technical progress, resource allocation, implementation issues, and new evidence from the literature or the field.

We used semi-structured interviews and focus groups with three stakeholder groups: (1) Patients with high blood pressure and other chronic diseases (*n* = 35), (2) primary care health professionals (general practitioners, professional nurses, staff nurses, pharmacists, allied health professionals, reception staff) (*n* = 12), (3) health care system service providers and subcontractors (provincial health systems managers (*n* = 5), third party providers of off-site pre-packaged repeat prescription services (*n* = 3)).

South Africa is a middle-income country with high levels of income inequality and a quadruple burden of disease (HIV/AIDS, maternal and child, non-communicable diseases, violence.) [32, 33] Health care is provided for most South Africans (over 80%) by publicly funded state run facilities the foundation of which are primary care facilities. Medical doctors and nurses (some who have the right to prescribe medications) staff facilities and provide diagnostic, and monitoring services; treatment including all medications is free for patients attending primary care (user fees for primary care were abolished in 1997.) [34] National guidelines for the treatment of high blood pressure exist and are regularly updated [35].There is an essential drugs list and medicines for high blood pressure available in primary care include thiazide and other diuretics, calcium channel blockers, ace-inhibitors, and beta-blockers. Patients maybe prescribed other anti-hypertensive agents like ARBs by specialists. Statins and Aspirin are also available [36].

With the stakeholders described above we explored the problem of high blood pressure and poor control in busy and resource constrained publically-funded primary care facilities. A range of problems were identified that were seen as barriers to providing optimal care and potential targets for intervention. These included organisation of care (failure of systems for referral between primary and secondary care and medication access), service provision (failure of clinicians to adhere to management guidelines), and patient-level factors such as sub-optimal self-management and treatment adherence. From discussion with the various stakeholder groups it emerged that patient-level factors resulting in failure to attend clinic appointments and collect and take medication regularly was both a major concern and a feasible and acceptable potential target for developing an intervention to improve blood pressure control. The underlying hypothesis was that facilitating communication between patients and the health care system might lead to changes in treatment adherence behaviour and improve health outcomes.

##### Use of mobile devices for intervention delivery

We framed the use of mobile phones as contextual tools that could deliver support messages when and where needed i.e. at times and places outside of a health care visit (ecological momentary intervention) [6]. We focused on using widely available existing communication protocols (for example short message service or SMS text messages) that are back-compatible (even the most basic device can send and receive text messages), and adapting participants’ existing technical skills to health specific behaviours rather than focusing on acquisition of new technical skills (for example by giving participants smart phones and asking them to use an app-based intervention).

##### Behaviour change theory

We explored a range of social cognition models and selected the I-CHANGE model that integrated multiple different elements (awareness, motivation, and action) which have been shown to be important in adherence and persistence with chronic medications [22, 37].

##### Behaviour change techniques

We used behaviour change theory to identify areas of belief or behaviour that might contribute to problems in collecting or taking medication. We then developed and refined the message content and mapped the messages to a common taxonomy of evidence-based behaviour change techniques [38].

##### Modelling (phase I)

We tested assumptions about the clarity, perceived usefulness and importance of individual text messages with stakeholder groups. To decide on the most appropriate tone and style of content delivery we tested individual SMS text messages using three different communication styles (directive, narrative vignette, or request). Stakeholders were asked when and how frequently adherence support messages should be sent. Messages that were unclear or ambiguous were modified; messages that were perceived by both patients and providers as not being useful or important were discarded. Patients’ thoughts and comments were also used to generate new content for new messages which were then again mapped to the taxonomy of behaviour change techniques and added to the message library. In addition, we engaged with the two local Community Advisory Boards (made up of community members and clinic patients who act as elected liaisons between the health facility and the community) who provided additional guidance and feedback on the intervention components and other study materials.

Using evidence from the literature alongside the findings from the semi-structured interviews of clinic and pharmacy staff at four representative primary care facilities in Cape Town the group agreed that in order to change adherence-related behaviours the intervention would need to,

- Remind patients about up-coming scheduled clinic appointments
- Provide relevant health-related information
- Help participants plan and organise various treatment adherence behaviours including medication collection and taking, diet, and exercise
- Support positive adherence-related behaviours
- Help navigate the health care system (e.g. what to do if the patient ran out of medications)

Table 2 gives examples of the SMS texts that were developed, mapped to the taxonomy of behaviour change techniques (along with definitions and message timing) [38].

**Table 2:** Behaviour change techniques used in the SMS text-messaging intervention

Behaviour change technique cluster^a^	Text message content^b^	Type of message^c^
Repetition and substitution		
Habit formation	Taking your medicine at the same time every day can help you remember to take your pills regularly.	Weekly
Behaviour substitution	Please remember, if you can’t make your MEDICINES TIME&DATE, send someone you trust to pick-up your pills. We need your clinic card and their identification document.	Weekly
Behavioural rehearsal/practice	Planning ahead (counting out tomorrow’s pills today) can help you remember to take your pills.	Weekly
Generalization of a target behaviour	Your good health is important. Please try to do more exercise. Activities that make you sweat or your heart beat faster are good for you.	Weekly
Natural consequences		
Health consequences	Please tell us (DR&PHARMACY) if you think your high blood pills are making you feel unwell. Ask us about common side effects of your pills.	Weekly
Salience of consequences	Please don’t give yours meds to people who are not prescribed them. Giving other people pills can endanger their health. Ask them to please come to the clinic.	Weekly
Anticipated regret	Did you know untreated high blood (when you don’t take your pills) puts you at risk for heart disease? Please take your pills as directed.	Weekly
Goals and planning		
Action planning	Ask someone you trust to help you remember to take your medicine as directed.	Weekly
Problem solving	Please remember to come back to clinic if you run out of medicine before your next date. You can come even if it is not your date.	Weekly
Commitment	Please remember your high blood is with you always. Work with [CLINIC NAME] to stay healthy. Keep your clinic dates & take your medicine as directed.	Weekly
Goal setting (outcome)	Please remember your next MEDICINE PICK-UP DATE is on [DAY][DD/MM/YY] at [00:00].	48 h prior to scheduled appointment
Behavioural contract	Please remember your high blood can’t be cured. To keep healthy Please keep on with your pills, come on your booked clinic dates, exercise & eat healthy food	Weekly
Review of behaviour goals	Thanks for picking up your meds. Keeping on your pills & attending on your correct dates helps us serve you better.	48 h post scheduled appointment
Social support		
Practical	Please be sure to tell the PHARMACY if you need to go away. We will give you a letter & extra pills so you won’t run out.	Weekly
General	Work with us to stay healthy. Learn about your condition & how to manage it. For more info ask us.	Weekly
Emotional	You are an important member of your community. Please keep trying with a healthy lifestyle. Please try to do more exercise.	Weekly

^a^Michie et al. [14]

^b^All text messages were signed off by a named health care provider

^c^Participants received one message per week, either a reminder to attend an up-coming appointment (48-h prior to scheduled appointment) or a message selected-at-random from the message library. Participants selected the time of day at which the message was sent, at trial recruitment

Patients and providers reported their thought that all people with high blood pressure could benefit from an intervention. Stakeholder groups reported disliking the idea of trying to target the intervention to particular patient groups, for example those with poor blood pressure control or those who only attend the clinic infrequently. Providers and health system managers cited concerns over the logistics of identifying such groups while patients reported concerns over perceptions of favouritism unrelated to illness severity. Patient groups expressed the opinion that “everyone with high blood pressure” should be offered the intervention and that people who didn’t want the intervention should be allowed to opt out.

Health care providers, particularly front-line staff preferred individual texts presented in a directive-style, for example, “You must take your medicine even if you feel well”. Reasons for this included the need to convey to patients the importance of the information being presented. In contrast all of the patient groups strongly preferred messages that were styled as polite requests, for example, “Please keep taking all your medicine even if you feel well”. Both groups were ambivalent about the use of narrative vignettes (for example “Busi in Langa: I bring my empties to clinic, then they can see I eat my pills right”.) Contextual aspects of the messages were also important (information specific to the clinic) as was the perceived authority of the message – messages signed off by a named provider were valued more highly by participants who felt they would be more likely to respond to such a message. Participants also reported that this was more important than using their name at the start of a message. Providers reported that it would be acceptable for senior staff at the facility to be named (i.e. sign off) in an SMS text as long as the messages were in line with Department of Health guidelines.

Individual SMS text messages are typically limited to 160 characters including spaces. We found that using short simple words was more acceptable to stakeholders than “textese” (a form of text-based slang using non-standard spelling and grammar). We minimised the use of contractions, using only “pls” for “please” and “thnks” for “thank you” and an abbreviation for the clinic name. All stakeholders groups reported on the value of having messages available in a variety of local languages though they did acknowledge that most people text in English (in part because of ambiguities in meaning that can arise from informal word shortening).

Participants reported that they valued the idea of being able to choose the time at which a message was sent so that it would not interfere with other commitments e.g. work or religious-activities. All stakeholders reported valuing the idea of a follow-up text message in the event a participant missed a scheduled appointment. On the basis of these discussions we decided to send follow-up messages to all participants thanking those who had attended on time and encouraging those who had not attended to please rebook their appointment. However, concerns were raised by providers and participants about the appropriate length of time between reminder messages and appointment dates so that people could make changes to their schedule or get in touch with the clinic to change their appointment. As a result, the messages were sent 48 h before and after a scheduled appointment.

Concerns about the potential costs of the intervention to the user were raised by all stakeholder groups. Specific concerns were raised about how to deliver an interactive intervention at little or no cost to the end-user. Solutions which have been used in other settings such as providing small amounts of credit to end-users to engage with an interactive system were rejected by health systems managers and sub-contractors due to concerns that the intervention would be too costly to deliver sustainably at scale. As a result of the telecommunications market in South Africa at the time it was not possible to use free-to-user short codes for interactive SMS text messagss.

##### Final interventions

The final interventions consisted of an adherence support intervention delivered by a weekly information-only (unidirectional) or interactive (bidirectional) SMS text message delivered at a time and in a language of the user’s choice. Messages were endorsed (signed off by a named provider) and contained content that was credible to both providers and patients and addressed a broad range of barriers to treatment adherence common in the local context. Reminder prompt text messages were sent 48 h before a scheduled clinic appointment (for a follow-up visit or to collect medication) with a follow-up message 48 h later either to thank participants for attending their appointment or to encourage them to rebook in the event of a missed appointment. To enable the system to be interactive we developed a system using free-to-user “Please Call Me” (or Call Me Back Code which is a service available on all local networks which allows a user to prompt someone else to call them) or missed-calls that enabled users to generate automated responses that allowed them to cancel or change their appointment, and change the time and language of the SMS text-messages.

By designing an intervention that was perceived in user-testing to be sent suitably frequently to keep users engaged (but not annoy them), contained content that was useful and could be trusted, and was phrased using polite and respectful language we felt the intervention would increase awareness and support motivation and actions to improve adherence to treatment for high blood pressure.

#### Modelling process and outcomes

**Fig. 2:**
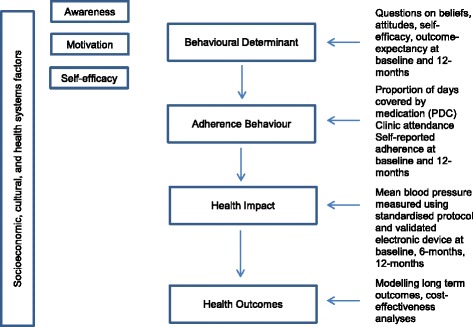
Hypothesised causal pathways and measures for evaluation for SMS text Adherence suppoRt (StAR) trial

We used a causal model to link theoretically relevant behavioural determinants to specific adherence related behaviours. We linked these to health impacts and outcomes along a hypothesised casual pathway [7, 8]. We used validated measures to assess important variables along the causal pathway. (See Fig. 2).

### Assessing feasibility and piloting methods

#### Availability and use of mobile phones among adults with chronic diseases attending primary care services in South Africa

To test assumptions about access to and use of mobile phones we conducted a cross-sectional survey among adult patients attending any one of five community health centres in the Western Cape Metro Health Districts for treatment of hypertension and other chronic diseases. These outpatient facilities provide comprehensive primary care services for people living in the surrounding areas.

At primary health care level, the service is based on prevention by educating people about the benefits of a healthy lifestyle. Every clinic has a staff member who has the skills to diagnose and manage chronic conditions, from young to elderly patients. Patients can see the same nurse for repeat visits if they come regularly on the hypertension, diabetes or asthma clinic day. Counselling, compliance, and health education are also part of usual care. The service is led by clinical nurse practitioners and supported by doctors.

Arrangements are made by the clinic to minimise patient travel (especially by the elderly) by prescribing supplies of drugs that last one to 3 months. Staff often facilitate the initiation of clubs and special support groups for people with chronic diseases. In this way, a patient can get more information on special care and health education pertaining to their condition.

These primary-level services are supported and strengthened by other levels of care, including acute and specialised referral hospitals. If complications arise, patients will be referred to the next level of care [39, 40].

The interviewer-administered questionnaire asked about socio-demographic factors, contact with the clinic, chronic diseases and treatment burdens and about access to and the use of mobile phones. We sampled consecutive consenting adults from outpatient services over a period of 6 weeks. A total of 127 willing and eligible adult patients completed the survey (see Table 3). Mean age (SD) was 53.3 (14) years, 73% were women, and two-thirds had at least some high-school education. Ninety percent of participants reported having regular (daily) access to a mobile phone and 80% reported that their phone was with them most or all of the time. Most participants did not share their phones (76%); women reported sharing more frequently than men (24% versus 13%). Most participants (76%) reported having registered their phone numbers in their name and that they had had the same mobile phone number for two or more years (63%).

**Table 3:** Selected characteristics of participants in the access to and use of mobile phones survey

		Male	Female	Total
Demographics				
*N*		30	97	127
Age		56 (13)	55 (12)	55 (12)
Level of formal education				
	Primary (%)	37	33	34
	High (%)	63	67	66
Employed (%)		37	25	27
Social grant access (if unemployed) (%)		84	77	78
Access to a mobile phone				
I have regular (daily) access to a mobile phone, *n*(%)		25 (83)	90 (93)	115 (90)
I own my own mobile phone, *n*(%)		21 (70)	75 (77)	96 (76)
I regularly share my phone with other people, *n*(%)		4 (13)	26 (27)	30 (24)
My phone is with me most or all of the time, *n*(%)		20 (67)	80 (82)	100 (79)
I have had the same mobile phone number for 2 or more years, *n*(%)		18 (60)	62 (64)	80 (63)
This phone number is registered to my identity number, *n*(%)		20 (67)	77 (79)	97 (76)
I would act to keep my same number even if my phone was lost or stolen or I changed provider, *n*(%)		23 (77)	85 (88)	108 (85)
Communication preferences				
I feel very confident using my phone to	Receive an SMS text, *n*(%)	17 (68)	64 (71)	81 (70)
	Send an SMS text, *n*(%)	14 (56)	49 (54)	63 (55)
	Make a call, *n*(%)	22 (88)	86 (95)	108 (94)
	Set a reminder, *n*(%)	14 (56)	38 (43)	52 (46)
	Add a contact, n(%)	17 (68)	53 (59)	70 (61)
	Send a “Please Call Me”, *n*(%)	18 (72)	63 (70)	81 (70)
	Cell-phone banking (USSD), *n*(%)	4 (16)	5 (6)	9 (8)
Attitudes towards text message intervention				
Perceptions of receiving a reminder to attend next clinic appointment	Helpful, *n*(%)	29 (97)	88 (91)	117 (92)
Perceptions of receiving a reminder to collect medications	Helpful, *n*(%)	30 (100)	90 (93)	120 (94)
Perceptions of receiving a reminder to take medications	Helpful, *n*(%)	26 (87)	85 (87)	111 (87)
Preferred contact type if clinic needs to get in touch	SMS text	14 (47)	75 (74)	89 (70)
	Phone call	14 (47)	18 (19)	32 (25)
	Other	2 (6)	4 (4)	6 (5)

70% of participants reported feeling very confident about using their phone to receive an SMS text messages, while fewer participants (55%) were as confident about sending SMS text messages. Most (70%) felt very confident about sending a “Please Call Me” a free service provided by South African telecommunications providers across all local networks. Fewer than 10% of participants reported knowing how to use unstructured supplementary service data (USSD) communication protocol services like mobile-phone banking.

When asked about preferred ways for the clinic to be in touch, more women (74%) than men (47%) preferred SMS text messages to phone calls or other methods like home visits. The majority of participants reported that they would find reminders to attend up-coming clinic appoints (92%), collect medications (94%), and take medications (87%) helpful.

#### Testing procedures

To optimise the intervention and test the technical systems responsible for message delivery we service tested the full intervention package with 19 patients recruited from patient-stakeholder focus groups. We tested the messages in the three languages most commonly used in Cape Town (English, Afrikaans, isiXhosa). Participants were contacted on a weekly basis by a researcher (experienced in qualitative methods) for a semi-structured interview on their experience of the intervention and the SMS text message delivery system. Suggestions were discussed with the intervention development team and changes were made where necessary.

#### Estimating recruitment and retention

In consultation with the local department of health we identified primary care health centres with high patient loads which might be suitable for a clinical trial to test the intervention. We visited each site to confirm the numbers of patients with high blood pressure using clinic registers, to map out patient flow through the clinic so we could operationalise trial procedures and identify potential challenges and barriers to implementation. One of the requirements for approval from the local department of health for research in public facilities is that normal activities are not interrupted. We therefore selected a health centre with a high caseload of patients with “chronic diseases of lifestyle” where we could recruit, screen, and enrol trial participants without interrupting the usual flow of patients through the clinic services.

We estimated we would be able to screen and recruit between 45 and 120 participants per week based on the functioning of the clinic and the experience of other local researchers [41]. We tested our capacity to recruit and screen participants using a clinical service offering blood pressure measurement to all patients attending the clinical service prior to the start of the trial. We tested trial registration and enrolment procedures including the receipt of an initial SMS text message at the time of enrolment. We collected detailed contact information on participants as well as the details of two next of kin (or similar) to maximise our chances of remaining in contact with participants for the duration of the trial. We monitored recruitment and retention on an ongoing basis.

#### Determining sample size

Adequately powered trials of the effects of m-health interventions on important clinical outcomes are required to develop the evidence base for these approaches [7, 8]. As blood pressure is strongly and directly related to mortality we selected change in mean systolic blood pressure at 12-months from baseline as our primary outcome (clinical). We selected medication adherence (behavioural) as a secondary outcome. We decided not to report these as co-primary outcomes. As there were no previous trials of the effect of adherence support delivered by SMS text on blood pressure measures we used data from published trials of behavioural interventions delivered using other methods to estimate sample size [42]. A decrease in systolic blood pressure of 5 mmHg is associated with clinically important reduction in the relative risk of stroke and coronary heart disease events [43]. Based on a study population similar to that expected for the trial population we used the standard deviation (SD) of systolic blood pressure (22.0 mmHg) to calculate the required sample size [44]. We proposed an intended target sample size of 1215 participants, allowing for 20% loss to follow-up, (at least 405 in each group) to detect an absolute mean difference in SBP of 5 mmHg (SD 22) at 12 months from baseline, with 90% power and 0.05 (two-sided) level of significance. We used an intention to treat (ITT) approach for all analyses.

### Evaluating a complex intervention

#### Assessing effectiveness

We decided that the most appropriate design to evaluate the effectiveness of adherence support via SMS text message would be a large single blind (concealed outcome assessment), individually randomised controlled trial. As the effect on clinical outcomes of an informational versus interactive system of SMS text messages was unclear from the published literature we decided to include two interventions; information-only SMS texts, and interactive SMS texts [8, 45]. To try to assess the effects of the behavioural intervention beyond receipt of an SMS text message from the clinic, we decided the control group would receive simple, infrequent text messages (less than one per month) related to the importance of ongoing trial participation [46]. Details of the intervention can be found in the TIDieR checklist (Table 4).

**Table 4:** Structured checklist systematically detailing intervention

TIDieR checklist for the SMS text Adherence suppoRt (StAR) intervention				
1.	Brief name	Control Group	Information-only SMS intervention	Interactive SMS intervention
2.	Rationale or theory	Mobile phones are contextual tools which could deliver an ecological momentary intervention [6]. Use SMS text’s because of their widespread availability and use, and we focused on adapting existing technical skills rather than acquiring new skills. We drew on an integrated theory of behaviour change [2], alongside with evidence-based behaviour change techniques (BCT) [3]; messages should be available in participants’ preferred language. As from the literature the relative effect on clinical outcomes of an informational versus interactive system of SMS text messages was unclear we included two intervention arms, one with information only SMS text’s, and one which included an interactive component.		
		Infrequent non-health related SMS texts sent to all participants, 1. Maintain participant interest in the trial. 2. Make it less clear who was getting which intervention 3. Exclude receipt of “any SMS” as effecting health-related behaviour		
			Information-only SMS intervention 1. Timely, relevant, personalised information designed to address common challenges to adherence 2. Content focused on BCT of goals and planning, repetition and substitution, social support, natural consequences 3. Unidirectional SMS text messages 4. Messages designed to be polite, direct, signed off by named provider	Interactive SMS intervention 1. Timely, relevant, personalised information designed to address common challenges to adherence 2. Content focused on BCT of goals and planning, repetition and substitution, social support, natural consequences 3. Bidirectional SMS text messages 4. Messages designed to be polite, direct, signed off by named provider
3.	Materials	Health information leaflet in preferred language		
4.	Procedures	1. Language and timing of messages selected by participant 2. Welcome SMS text 3. Happy birthday SMS text 4. Non-health related SMS text message sent at 6-weekly intervals (randomly selected)	1. Language and timing of messages selected by participant 2. Welcome SMS text 3. Happy birthday SMS text 4. Non-health related SMS text message sent at 6-weekly intervals (randomly selected) 5. Weekly SMS text message, randomly selected from library (with rule that ensured messages were not repeated) 6. SMS text message reminder to attend scheduled clinic appointment 48 h prior to date 7. SMS text message to either thank participant for attending appointment or alert participants about a missed appointment 48 h post date	1. Language and timing of messages selected by participant 2. Welcome SMS text 3. Happy birthday SMS text 4. Non-health related SMS text message sent at 6-weekly intervals (randomly selected) 5. Interactive-SMS to check timing and language of messages was acceptable (automated system to make change if required) 6. Weekly SMS text message, randomly selected from library (with rule that ensured messages were not repeated) 7. Interactive-SMS to remind participant of up-coming appointment and offer to reschedule if date no-longer convenient (48 h prior to appointment date) 8. Interactive-SMS thanking participant for attending appointment or offer to reschedule a missed appointment 48 h post date) 9. Interactive-SMS to trouble shoot common problems at the health facility (long queues, lost folders)
5.	Intervention provider	Automated SMS text delivery platform using open-source software		
6.	Modes of delivery	Intervention delivered via 160 character SMS text sent to individual participant’s own handset		
7.	Location where intervention occurred	Outside of health care facility, where ever participant and their phone were located (real world)		
8.	Number of times intervention was delivered over what time period	SMS text message sent about once every 6 weeks for 12-months	SMS text message sent weekly for 12-months	SMS text message sent weekly for 12-months (with follow-up messages generated through user initiated dialogue)
9.	What, why, when, how intervention was personalised or adapted	1. Language and timing of messages selected by participant 2. Date of birth recorded for birthday message	1. Language and timing of messages selected by participant 2. Date of birth recorded for birthday message 3. Personalised timing of appointment reminders based on prospectively routinely collected computerised appointment data	1. Language and timing of messages selected by participant 2. Date of birth recorded for birthday message 3. Interactive-SMS to check timing and language of messages was acceptable (if not automated SMS-dialogue to change either language or timing) 4. Personalised timing of appointment reminders based on prospectively routinely collected computerised appointment data 5. Regular interactive-SMS to enable rescheduling of up-coming or missed appointments, and to troubleshoot common challenges at the health facility
10.	Modifications during the trial	Nil	Nil	Nil
11.	Planned intervention delivery	SMS text messages were sent using an automated system independent of trial and clinical staff. Participants were told that not everyone will be receiving the exact same messages. Participants will also be asked not to share the SMS text messages with others. Intervention fidelity was checked by confirming receipt at least of an initial “Welcome” SMS text message for all enrolled trial participants prior to randomisation. Message delivery reports were monitored throughout the trial to check the intervention was being delivered as planned. Messages not delivered (network unavailable etc) were resent up to three times		
12.	Actual intervention delivery	8277 individual SMS text messages over 12-month period (457 participants)	40,333 individual SMS text messages over 12-month period (458 participants)	41,450 individual SMS text messages over 12-month period (458 participants)

The trial is registered with the South African National Clinical Trials Register number (SANCTR DOH-27-1212-386; 28/12/2012); Pan Africa Trial Register (PACTR201411000724141; 14/12/2013); ClinicalTrials.gov (NCT02019823; 24/12/2013).

#### Understanding change processes

##### Implementation. Fidelity assessment

SMS text messages were sent using an automated system independent of trial and clinical staff. Participants were informed that not everyone would be receiving the exact same messages. Participants were also asked not to share the SMS text messages with others. Intervention fidelity was ensured by confirming the receipt at least of an initial “Welcome” SMS text message for all enrolled trial participants prior to randomisation. Message delivery reports were monitored throughout the trial to check the intervention was being delivered as planned. In addition, we also set up a system of sentinel-phones (using the five most common entry-level handsets in South Africa) registered and allocated to receive messages in the same way as trial participants.

The trial interventions were delivered separately from the health care workers providing usual clinical care for participants. For each anticipated study visit (enrolment, 6-month follow-up, 12-month follow-up) standardised protocols were used. Structured logs were used to record detailed information for any interactions between trial staff and participants outside of expected study visits.

##### Contextual factors

In the final stages of the trial we conducted an independent process evaluation to explore the implementation of the intervention, contextual factors, and potential mechanisms of action. We employed a qualitative design using focus groups and in-depth interviews. The findings from the evaluation have been reported separately [47].

#### Cost-effectiveness analyses

We collected information on the costs of developing and delivering the intervention. The findings from this analysis will be reported separately.

### Implementation and beyond

#### Dissemination

The potential to accumulate evidence of effectiveness and to identify the “active components” in successful m-health interventions depends in part on replication of successful interventions across settings and refining interventions (adding or subtracting elements) using evidence of behaviour change. To facilitate use and adaptation of our intervention we have used recommendations for reporting intervention development to ensure we have described the intervention and its delivery in sufficient detail [13]. We have registered the trial and published the trial protocol [48], and we will publish the trial results (using CONSORT reporting guidelines) in an open access journal. We have also published the findings from the process evaluation [in press]. We have reported findings to participants, health care workers, policy makers, and funders.

#### Surveillance, monitoring, and long-term follow up

We obtained permission to collect routine health data (dispensing and adherence data) from trial participants for a period of 6 months after the trial ended to explore for persistence of effects of the intervention (if any).

## Discussion

### Main findings

Using the MRC Framework was feasible in a low-resource multi-lingual setting. The adoption of the framework enabled us to develop a theory- and evidence-based intervention; to specify a proposed causal pathway to modify adherence behaviour and clinical outcomes; to test and refine the intervention delivery system; to design a randomised evaluation of the intervention; and to test and evaluate proposed study procedures.

### What is already known on this topic

Mobile devices are a promising approach for delivering health interventions [7, 8]. Replication of study findings is hampered by the lack of adequate description of specific intervention components and their theoretical basis [13–15]. A number of frameworks have been proposed in the health and technology fields to aid the design of technology-based interventions [16, 49–51]. The 2008 MRC Framework has been used to design and evaluate interventions across disciplines which suggest its flexible, comprehensive, non-linear, iterative approach may be applicable to the design and evaluation of m-health interventions [15, 17–20].

### What this study adds

This paper shows how the framework can be operationalised for an m-health intervention by explicitly mapping the activities, development, and testing to the stages of the 2008 MRC framework. We have also included detailed descriptions of the various aspects of the intervention and its delivery, reported in-line with TIDieR guidelines, which will enable comparison with other m-health interventions and support development of new interventions. Lastly, we have demonstrated that it is feasible and beneficial to use this approach in a multi-lingual low-resource setting.

### Limitations of this study/framework

Sufficient time and resources need to be available to apply the Framework and benefit from the iterative development process and from testing of study-related procedures. For example, it took us several months longer than anticipated to complete the intervention development and testing in part because in resource constrained settings like the public health facilities in South Africa it can be challenging for frontline service staff to find time to engage in intervention design activities (interviews, discussions, message library review.)

Whilst the intervention development work was carried out at several sites the clinical trial was at a single-site. In future, we will engage in both development and testing across sites to tease out factors that are common and unique for specific mHealth interventions.

Lastly, attention also needs to be given to field testing of recruitment and retention strategies as there are many instances where trials of mHealth interventions in similar settings are inconclusive because of poor recruitment and high rates of loss to follow-up [52, 53].

## Conclusions

The MRC Framework can be successfully applied to develop and evaluate m-health interventions in a multi-lingual resource-constrained setting. Detailed descriptions of the development process, the intervention and its delivery may advance the evidence-base for m-health interventions, enabling comparison, adaption, and development of interventions.

## Abbreviations

MRC: Medical Research Council
StAR: SMS text Adherence suppoRt trial

## References

[1] Lim SS, Vos T, Flaxman AD, Danaei G, Shibuya K, Adair-Rohani H (2012). A comparative risk assessment of burden of disease and injury attributable to 67 risk factors and risk factor clusters in 21 regions, 1990-2010: a systematic analysis for the global burden of disease study 2010. Lancet.

[2] Lewington S, Clarke R, Qizilbash N, Peto R, Collins R, Prospective Studies Collaboration (2002). Age-specific relevance of usual blood pressure to vascular mortality: a meta-analysis of individual data for one million adults in 61 prospective studies. Lancet.

[3] Burnier M (2006). Medication adherence and persistence as the cornerstone of effective antihypertensive therapy. Am J Hypertens.

[4] Viswanathan M, Golin CE, Jones CD, Ashok M, Blalock SJ, Wines RCM (2012). Interventions to improve adherence to self-administered medications for chronic diseases in the United States: a systematic review. Ann Intern Med.

[5] Gwadry-Sridhar FH, Manias E, Lal L, Salas M, Hughes DA, Ratzki-Leewing A (2013). Impact of interventions on medication adherence and blood pressure control in patients with essential hypertension: a systematic review by the ISPOR medication adherence and persistence special interest group. Value Health.

[6] Heron KE, Smyth JM (2010). Ecological momentary interventions: incorporating mobile technology into psychosocial and health behaviour treatments. Br J Health Psychol.

[7] Free C, Phillips G, Galli L, Watson L, Felix L, Edwards P (2013). The effectiveness of mobile-health technology-based health behaviour change or disease management interventions for health care consumers: a systematic review. PLoS Med.

[8] Beratarrechea A, Lee AG, Willner JM, Jahangir E, Ciapponi A, Rubinstein A (2014). The impact of mobile health interventions on chronic disease outcomes in developing countries: a systematic review. Telemed J E Health.

[9] Lester RT, Ritvo P, Mills EJ, Kariri A, Karanja S, Chung MH (2010). Effects of a mobile phone short message service on antiretroviral treatment adherence in Kenya (WelTel Kenya1): a randomised trial. Lancet.

[10] Mbuagbaw L, Thabane L, Ongolo-Zogo P, Lester RT, Mills EJ, Smieja M, et al. The Cameroon Mobile Phone SMS (CAMPS) Trial: A Randomized Trial of Text Messaging versus Usual Care for Adherence to Antiretroviral Therapy. PLoS ONE. 2012;7(12):e46909. 10.1371/journal.pone.0046909 10.1371/journal.pone.0046909 PMC3516507 23236345

[11] Yasmin F, Banu B, Zakir SM, Sauerborn R, Ali L, Souares A (2016). Positive influence of short message service and voice call interventions on adherence and health outcomes in case of chronic disease care: a systematic review. BMC Med Inform Decis Mak.

[12] Adler AJ, Martin N, Mariani J, Tajer CD, Owolabi OO, Free C, The Cochrane Collaboration (2017). Mobile phone text messaging to improve medication adherence in secondary prevention of cardiovascular disease. Cochrane database of systematic reviews.

[13] Hoffmann TC, Glasziou PP, Boutron I, Milne R, Perera R, Moher D (2014). Better reporting of interventions: template for intervention description and replication (TIDieR) checklist and guide. BMJ.

[14] Michie S, Brown J, Geraghty AWA, Miller S, Yardley L, Gardner B (2012). Development of StopAdvisor. Transl Behav Med.

[15] Lakshman R, Griffin S, Hardeman W, Schiff A, Kinmonth AL, Ong KK (2014). Using the Medical Research Council framework for the development and evaluation of complex interventions in a theory-based infant feeding intervention to prevent childhood obesity: the baby milk intervention and trial. J Obes.

[16] Nhavoto JA, Grönlund Å, Chaquilla WP (2015). SMSaúde: design, development, and implementation of a remote/mobile patient management system to improve retention in care for HIV/AIDS and tuberculosis patients. JMIR MHealth UHealth.

[17] Modi D, Gopalan R, Shah S, Venkatraman S, Desai G, Desai S (2015). Development and formative evaluation of an innovative mHealth intervention for improving coverage of community-based maternal, newborn and child health services in rural areas of India. Glob Health Action.

[18] Craig P, Dieppe P, Macintyre S, Michie S, Nazareth I, Petticrew M. Developing and evaluating complex interventions: the new Medical Research Council guidance. BMJ. 2008;337:a1655. 10.1136/bmj.a1655. 10.1136/bmj.a1655 PMC2769032 18824488

[19] Paul G, Smith SM, Whitford D, O’Kelly F, O’Dowd T (2007). Development of a complex intervention to test the effectiveness of peer support in type 2 diabetes. BMC Health Serv Res.

[20] Smith SM, Murchie P, Devereux G, Johnston M, Lee AJ, Macleod U (2012). Developing a complex intervention to reduce time to presentation with symptoms of lung cancer. Br J Gen Pract.

[21] Moore GF, Audrey S, Barker M, Bond L, Bonell C, Hardeman W (2015). Process evaluation of complex interventions: Medical Research Council guidance. BMJ.

[22] Nieuwlaat R, Wilczynski N, Navarro T, Hobson N, Jeffery R, Keepanasseril A (2014). Interventions for enhancing medication adherence. Cochrane Database Syst Rev.

[23] Márquez Contreras E, de la Figuera von Wichmann M, Gil Guillén V, Ylla-Catalá A, Figueras M, Balaña M (2004). Effectiveness of an intervention to provide information to patients with hypertension as short text messages and reminders sent to their mobile phone (HTA-Alert). Atencion Primaria.

[24] Márquez Contreras E, Vegazo García O, Martel Claros N, Gil Guillén V, de la Figuera v, Wichmann M, Casado Martínez JJ (2005). Efficacy of telephone and mail intervention in patient compliance with antihypertensive drugs in hypertension. ETECUM-HTA study. Blood Press.

[25] Morikawa N, Yamasue K, Tochikubo O, Mizushima S (2011). Effect of salt reduction intervention program using an electronic salt sensor and cellular phone on blood pressure among hypertensive workers. Clin Exp Hypertens.

[26] de Jongh T, Gurol-Urganci I, Vodopivec-Jamsek V, Car J, Atun R (2012). Mobile phone messaging for facilitating self-management of long-term illnesses. Cochrane Database Syst Rev.

[27] Buhi ER, Trudnak TE, Martinasek MP, Oberne AB, Fuhrmann HJ, McDermott RJ. Mobile phone-based behavioural interventions for health: a systematic review. Health Educ J. 2012; 10.1177/0017896912452071.

[28] Carrasco MP, Salvador CH, Sagredo PG, Márquez-Montes J, González de Mingo MA, Fragua JA (2008). Impact of patient-general practitioner short-messages-based interaction on the control of hypertension in a follow-up service for low-to-medium risk hypertensive patients: a randomized controlled trial. IEEE Trans Inf Technol Biomed.

[29] Blasco A, Carmona M, Fernández-Lozano I, Salvador CH, Pascual M, Sagredo PG (2012). Evaluation of a telemedicine service for the secondary prevention of coronary artery disease. J Cardiopulm Rehabil Prev.

[30] Logan AG, Irvine MJ, McIsaac WJ, Tisler A, Rossos PG, Easty A (2012). Effect of home blood pressure telemonitoring with self-care support on uncontrolled systolic hypertension in diabetics. Hypertension.

[31] McKinstry B, Hanley J, Wild S, Pagliari C, Paterson M, Lewis S (2013). Telemonitoring based service redesign for the management of uncontrolled hypertension: multicentre randomised controlled trial. BMJ.

[32] Mayosi BM, Lawn JE, van Niekerk A, Bradshaw D, Abdool Karim SS, Coovadia HM (2012). Health in South Africa: changes and challenges since 2009. Lancet.

[33] Ataguba JE-O, Day C, McIntyre D. Explaining the role of the social determinants of health on health inequality in South Africa. Glob Health Action. 2015;8:28665. 10.3402/gha.v8.28865. 10.3402/gha.v8.28865 PMC4575416 26385543

[34] Wilkinson D, Gouws E, Sach M, Karim SS (2001). Effect of removing user fees on attendance for curative and preventive primary health care services in rural South Africa. Bull World Health Organ.

[35] Seedat Y, Rayner B, Veriava Y (2014). South African hypertension practice guideline 2014. Cardiovasc J Afr.

[36] Essential Drugs Programme (EDP) [Internet]. [cited 2017 Jun 21]. Available from: http://www.health.gov.za/index.php/essential-drugs-programme-edp

[37] de Josselin de Jong S, Candel M, Segaar D, Cremers H-P, de Vries H. Efficacy of a web-based computer-tailored smoking prevention intervention for Dutch adolescents: randomized controlled trial. J Med Internet Res. 2014;16(3):e82. 10.2196/jmir.2469. 10.2196/jmir.2469 PMC3978560 24657434

[38] Michie S, Richardson M, Johnston M, Abraham C, Francis J, Hardeman W (2013). The behavior change technique taxonomy (v1) of 93 hierarchically clustered techniques: building an international consensus for the reporting of behavior change interventions. Ann Behav Med.

[39] Healthcare 2030: A Future Health Service for the Western Cape [Internet]. Western Cape Government. [cited 2017 Jun 21]. Available from: https://www.westerncape.gov.za/news/healthcare-2030-future-health-service-western-cape

[40] Chronic Care [Internet]. Western Cape Government. [cited 2017 Jun 21]. Available from: https://www.westerncape.gov.za/service/chronic-care

[41] Stewart S, Carrington MJ, Pretorius S, Ogah OS, Blauwet L, Antras-Ferry J (2012). Elevated risk factors but low burden of heart disease in urban African primary care patients: a fundamental role for primary prevention. Int J Cardiol.

[42] Schroeder K, Fahey T, Ebrahim S. Interventions for improving adherence to treatment in patients with high blood pressure in ambulatory settings. Cochrane Database Syst Rev. 2004. http://onlinelibrary.wiley.com/doi/10.1002/14651858.CD004804/abstract. 10.1002/14651858.CD004804 PMC9036187 15106262

[43] Collins R, Peto R, MacMahon S, Hebert P, Fiebach NH, Eberlein KA (1990). Blood pressure, stroke, and coronary heart disease. Part 2, short-term reductions in blood pressure: overview of randomised drug trials in their epidemiological context. Lancet.

[44] Tibazarwa K, Ntyintyane L, Sliwa K, Gerntholtz T, Carrington M, Wilkinson D (2009). A time bomb of cardiovascular risk factors in South Africa: results from the heart of Soweto study ‘heart awareness days’. Int J Cardiol.

[45] Finitsis DJ, Pellowski JA, Johnson BT (2014). Text message intervention designs to promote adherence to antiretroviral therapy (ART): a meta-analysis of randomized controlled trials. PLoS One.

[46] Free C, Knight R, Robertson S, Whittaker R, Edwards P, Zhou W (2011). Smoking cessation support delivered via mobile phone text messaging (txt2stop): a single-blind, randomised trial. Lancet.

[47] Leon N, Surender R, Bobrow K, Muller J, Farmer A (2015). Improving treatment adherence for blood pressure lowering via mobile phone SMS-messages in South Africa: a qualitative evaluation of the SMS-text Adherence SuppoRt (StAR) trial. BMC Fam Pract.

[48] Bobrow K, Brennan T, Springer D, Levitt NS, Rayner B, Namane M (2014). Efficacy of a text messaging (SMS) based intervention for adults with hypertension: protocol for the StAR (SMS Text-message Adherence suppoRt trial) randomised controlled trial. BMC Public Health.

[49] Glasgow RE, Vogt TM, Boles SM (1999). Evaluating the public health impact of health promotion interventions: the RE-AIM framework. Am J Public Health.

[50] Spoth R, Rohrbach LA, Greenberg M, Leaf P, Brown CH, Fagan A (2013). Addressing Core challenges for the next generation of type 2 translation research and systems: the translation science to population impact (TSci impact) framework. Prev Sci.

[51] Crosby R, Noar SM (2011). What is a planning model? An introduction to PRECEDE-PROCEED. J Public Health Dent.

[52] Rubinstein A, Miranda JJ, Beratarrechea A, Diez-Canseco F, Kanter R, Gutierrez L (2016). Effectiveness of an mHealth intervention to improve the cardiometabolic profile of people with prehypertension in low-resource urban settings in Latin America: a randomised controlled trial. Lancet Diabetes Endocrinol.

[53] Lau YK, Cassidy T, Hacking D, Brittain K, Haricharan HJ, Heap M (2014). Antenatal health promotion via short message service at a midwife obstetrics unit in South Africa: a mixed methods study. BMC Pregnancy Childbirth.

